# Appearance of malignant melanoma after a non-cutaneous cancer diagnosis

**DOI:** 10.3332/ecancer.2013.315

**Published:** 2013-05-07

**Authors:** Ugo Bottoni, Rita Clerico, Giovanni Paolino, Marina Ambrifi, Cecilia Luci, Paola Corsetti, Stefano Calvieri

**Affiliations:** 1 University Magna Graecia, V.le Europa, 88100 Catanzaro, Italy; 2 Clinica Dermatologica, Dipartimento di Medicina Interna e Specialità Mediche, University of Rome, La Sapienza, Viale del Policlinico, Rome, Italy

**Keywords:** melanoma, renal cell carcinoma, non-cutaneous malignancies, thyroid cancer, multiple primary malignancies

## Abstract

**Background:**

The aim of this study is to find the associations between malignant melanoma (MM) and other non-cutaneous malignancies and to see whether there are possible correlations between them.

**Methods:**

We analysed a sample of 1720 patients collected by our melanoma database, to identify patients with both MM and non-cutaneous primary cancer (NCC). The incidence rate (IR) included in our database was calculated as the ratio between the observed patients with NCC and those with MM.

**Results:**

A total of 74 patients, with both NCC and MM, were included in our analysis, corresponding to 4.30% of patients with MM present in our melanoma database. After breast cancer (24.3%; IR = 1:4), the most common malignancies were lymphomas (14.8%; IR = 1:4), renal cell carcinoma (13.5%; IR = 1:7), thyroid cancer (9.4%; IR = 1:11), and prostatic carcinoma (8.1%; IR = 1:12), followed by other cancers. Among patients with lymphomas, most patients (72.7%) had a non-Hodgkin lymphoma. Our study shows a high coexistence of multiple malignancies in patients with MM.

**Conclusion:**

Although we cannot definitively confirm a true association between non-skin cancers and MM, we believe that there are sufficient links for further investigation in order to identify new aetiological factors and therapeutic targets for these cancers.

## Introduction

The occurrence of multiple primary malignancies in the same patient is a well-recognized phenomenon. The increased incidence of subsequent non-cutaneous primary cancers (NCCs) in patients with cutaneous malignant melanoma (MM) is well documented [1].

Although in the literature a significant excess of NCC after an MM has been reported, an excess of MM after an NCC is much less frequently highlighted. Currently, there have been no clinicopathological reviews of such cases [2, 3].

We report the results obtained from an analysis of 1720 patients affected by MM over a period of 15 years.

## Materials and methods

We analysed a sample of 1720 patients from our melanoma database, to identify patients with both MM and NCCs.

All NCCs, diagnosed during the initial MM staging, were considered synchronous. The clinical presentations were defined as asymptomatic for tumours that were discovered at any radiologic examination. The median follow-up of the entire cohort was 84 months.

The incidence rate (IR) was calculated as the ratio between the observed patients with NCC and with MM, and was included in our database. A 95% confidence interval was also performed for the entire cohort.

## Results

Our analysis of 74 patients, with both NCC and MM, totaled 4.30% of patients with MM present in our melanoma database. Epidemiological analyses have reported a higher prevalence of female patients (*n *= 45) than male patients (*n *= 29). The median age of the entire cohort was of 56.5 years (ranging from 26 to 76) for female patients and 58 years (ranging from 25 to 74) for male patients (Table 1).

Regarding the primary melanoma thickness, 35 patients removed lesions ≤1.00 mm, while 29 patients ≥1.01 mm (ranging between 0.12 and 9.5 mm). According to the seventh American Joint Committee on Cancer (AJCC) for melanoma [4], 44 patients were in stage I, 16 in stage II, and nine in stage III. Two patients presented an MM with unknown primary. While, regarding NCC, 26 patients (35.13%) were in stage I at diagnosis, 30 patients (40.54%) were in stage II, 15 patients (20.3%) in stage III, and only three patients (4.1%) in stage IV.

After breast cancer (24.3%; IR = 1: 4), the most common malignancies were lymphomas (14.8%; IR = 1:4), renal cell carcinoma (13.5%; IR = 1:7), thyroid cancer (9.4%; IR = 1:11), and prostatic carcinoma (8.1%; IR = 1:12), followed by other cancers as reported in Table 2.

Among patients with lymphomas, most patients (72.7%) had a non-Hodgkin lymphoma. Among the non-Hodgkin lymphomas, four patients (50%) presented a follicular lymphoma, two patients (25%) a large B-cell lymphoma, one patient (12.5%) a small lymphocytic lymphoma, and one patient (12.5%) a mantle cell lymphoma.

Three female patients and two male patients showed a positive history for immunological disease such as rheumatoid arthritis, psoriatic arthritis, and autoimmune thyroiditis.

In the 66 patients analysed, an MM occurred after an NCC diagnosis, while in seven patients, an NCC was found after an MM diagnosis. In one patient, a renal cell carcinoma was found during MM staging and as a result it was considered synchronous.

No patients in our cohort showed a positive family history for MM or other syndromes that could justify the onset of multiple malignancies. B-RAF mutation was positive in one patient (1.4%) with asynchronous renal cell carcinoma.

For treatment a radiotherapy regimen was performed in six patients, a combined chemo and radiotherapy treatment was performed in 19 patients, while a chemotherapy treatment alone was performed in 45 patients. For the remaining patients, treatment was not performed because it was not provided or because the patients refused any treatment.

The most common MM risk factors observed in our cohort were the same for the general population (I and II Fitzpatrick’s photo type, high number of nevi, intermittent and intense sun exposure and sunburn at a young age), as well as an eventual immune system deficiency history (as reported in nine patients that had also had a bone marrow transplant). In fact, seven patients affected by lymphomas showed a white blood cell count of ≤4.500/μl at time of first visit.

The overall survival rate of the entire cohort was of 54 months (ranging from six to 144 months); in 77.7% of cases, this was connected to NCC, while in only 22.3%, it was connected to MM.

## Discussion

The coexistence of multiple cancers in the same patient has been widely documented [2, 3, 5–7]. It is well established that patients with MM are at higher risk than the general population for developing a secondary primary tumour. However, the development of MM subsequently to NCCs is less documented.

The 4.3% of patients in our MM database also presented with an NCC. The most common cancers were breast cancer, lymphomas, and renal cell carcinoma. However, considering the high incidence of breast and prostatic cancers [8] in the general population, and the increased risk of developing a secondary malignancy in patients with haematologic disorders who received immunosuppressive therapies [6], the percentage (13.5%) of renal cell carcinoma and thyroid carcinoma out of our cohort can be emphasized.

The absence of familial MM in our cohort can also be explained by the simultaneous absence of pancreatic carcinoma. In the literature, the presence of pancreatic cancer in MM prone families has been consistently associated with an increased frequency of CDKN2A mutations [9, 10].

Like MM, the incidence of renal cell carcinoma has increased in the last few decades and different studies have found an association between these two malignancies [3–5]. In the literature, an excess of renal cell carcinoma and NCC after an MM diagnosis is often reported. However, in our analysis and in contrast with recent studies, 66 (89.18%) patients developed an NCC (including renal cell carcinoma) before an MM diagnosis [1, 3, 5–7].

Although the simultaneous occurrence of MM and NCC may be coincidental, there are several plausible links. In particular, recent studies support a genetic predisposition of coexisting MM and kidney cancer in the same patient, such as microphthalmia-associated transcription factor, CDKN2A and B-RAF [11–15].

Recently, B-RAF mutations have also been identified in thyroid carcinoma [16]; in this regard, the high number of thyroid carcinomas (9.4%) in our sample should be mentioned (Table 2).

Furthermore, in our cohort, there was a high prevalence of multiple malignancies (60.8%) in female patients. Considering the higher incidence of immunological diseases in female than in male patients [17], it can be hypothesized that immunological mechanisms predispose these patients to multiple malignancies. In our cohort, five patients (6.75%) showed a positive history for immunological diseases with previous immunomodulating treatments.

Finally the pre-existence of a cancer can alter the human immune system and facilitate the possible occurrence of a secondary malignancy; this can be related to the disease’s course or to the therapies.

Considering recent studies that have highlighted a possible role of virus-like particles in MM cells and also oncolytic viruses in several malignancies [18–20], a possible viral role in the onset of NCC associated with MM cannot be ruled out.

In conclusion, our study shows a high coexistence of multiple malignancies in patients with MM. Although we cannot definitively confirm a true association between NCC and MM, we believe that there are sufficient links for further investigation in order to identify new aetiological factors and therapeutic targets for these cancers.

## Figures and Tables

**Table 1:** Gender and age-adjusted incidence of non-cutaneous cancers (NCCs) in our cohort.

NCC	AGE INCIDENCE	GENDER
Breast	0−49: 3	3 F : 0 M
	50−69: 8	8 F : 0 M
	≥70: 7	7 F : 0 M
Lymphomas	0−49: 1	1 F : 0 M
	50−69: 2	0 F : 2 M
	≥70: 8	3 F : 5 M
Kidney	0−49: 3	3 F : 0 M
	50−69: 4	2 F : 2 M
	≥70: 3	3 F : 0 M
Thyroid	0−49: 2	2 F : 0 M
	50−69: 1	1 F : 0 M
	≥70: 4	4 F : 0 M
Prostate	0−49: 0	0 F : 0 M
	50−69: 1	0 F : 1 M
	≥70: 5	0 F : 5 M
Larynx	0−49: 0	0 F : 0 M
	50−69: 1	1 F : 0 M
	≥70: 4	2 F : 2 M
Bladder	0−49 : 0	0 F : 0 M
	50−69 : 2	0 F : 2 M
	≥70 : 1	0 F : 1 M
Stomach	0−49 : 0	0 F : 0 M
	50−69 : 0	0 F : 0 M
	≥70 : 3	0 F : 3 M
Lung	0−49 : 0	0 F : 0 M
	50−69 : 1	0 F : 1 M
	≥70 : 2	1 F : 1 M
Colon−rectum	0−49 : 0	0 F : 0 M
	50−69: 0	0 F : 0 M
	≥ 70: 2	1 F : 1 M
Others	0−49 : 1	1 F : 0 M
	50−69: 2	1 F : 1 M
	≥70: 3	1 F : 2 M

**Table 2:** Non-cutaneous cancers (NCCs) and relative incidence rate (IR) present in our cohort.

NCC	N°	%	IR	95% CI	GP (%)
Breast	18	24.32	1:4	0.1–0.3	13.97
Lymphomas	11	14.86	1:4	0.1–0.3	4.81
Kidney	10	13.51	1:7	0.06–0.2	3.95
Thyroid	7	9.45	1:11	0.03–0.2	3.44
Prostate	6	8.10	1:12	0.02–0.2	14.75
Larynx	5	6.75	1:12	0.02–0.1	0.75
Bladder	3	4.01	1:25	0.01–0.1	4.48
Stomach	3	4.01	1:25	0.01–0.1	1.30
Lung	3	4.01	1:25	0.01–0.1	13.79
Colon-Rectum	2	2.70	1:37	0.01–0.1	8.78
Others	6	8.10	1:12	0.02–0.1	29.98

95% CI indicates 95% confidence interval.

GP (%) indicates the percentage of relative malignancies among general population.
